# Case report and diagnostic implications of misdiagnosis of pericardial myxoid liposarcoma by multimodal imaging

**DOI:** 10.3389/fcvm.2025.1685844

**Published:** 2025-10-09

**Authors:** Juan Wang, Xi-Jun Zhu, Li-Xia Sun, Yu-Ling Song, Lai Wu, Yun-Long Cao, Xin-Yu Xue, Dong Wang, Qian Liu

**Affiliations:** ^1^ Department of Cardiology, Binzhou Medical University Hospital, Binzhou, China; ^2^ Department of Pathology, Binzhou Medical University Hospital, Binzhou, China; ^3^ Department of Endocrinology, Binzhou Medical University Hospital, Binzhou, China; ^4^ Department of Ultrasound, Binzhou Medical University Hospital, Binzhou, China

**Keywords:** CMR, echocardiography, cardiac mass, pericardial tumor, myxoid liposarcoma

## Abstract

Myxoid liposarcoma (MLPS) typically occurs in the extremities. Pericardial involvement is exceptionally rare and usually indicates metastatic disease. Because of their non-specific imaging features, such lesions are often mistaken for benign tumors, leading to diagnostic errors. This report describes the case of a 69-year-old woman who presented with chest tightness and had a history of gluteal MLPS. Multimodal imaging (CT, ultrasound, MRI) revealed a pericardial mass. Although features such as well-circumscribed margins and delayed contrast filling suggested a benign-appearing lesion rather than enabling a definitive diagnosis, the clinical history of the patient strongly favored metastatic MLPS. Imaging alone could not provide a definitive diagnosis, highlighting the challenge posed by overlapping features between benign and malignant cardiac masses. The final diagnosis relied on histopathology and molecular testing. Postoperative immunohistochemistry revealed a spindle cell tumor with myxoid stroma (S-100 negative, MDM2 weakly positive). Molecular pathology confirmed the diagnosis by detecting the FUS-DDIT3 fusion gene, establishing metastatic MLPS. This case underscores the critical limitations of imaging in reaching a definitive diagnosis and emphasizes that accurate classification necessitates integration with histopathological and molecular analyses. An optimized diagnostic strategy should incorporate a comprehensive review of clinical history—especially any prior sarcoma—maintain heightened vigilance for overlapping imaging features of rare sarcomas in atypical locations, and include molecular pathology to effectively prevent misdiagnosis.

## Introduction

Myxoid liposarcoma (MLPS) is a subtype of malignant soft tissue sarcoma, accounting for approximately 5% of all cases (1, 2). It typically arises in the deep soft tissues of the extremities, while its primary or metastatic involvement of the pericardial cavity is exceedingly rare. MLPS is metastatic in approximately 14%–33% of cases, most frequently to soft tissue, bone, and abdominal organs. Cardiac involvement, however, is exceedingly rare, and pericardial involvement (whether primary or metastatic) is even more exceptional (3–5). In the literature, about 35 cases of cardiac metastases have been reported, with only nine involving the pericardium. A more recent systematic review identified 46 cardiac MLPS metastases, of which 16 (34%) affected the pericardium. While primary sites are typically in the extremities (e.g., thigh), retroperitoneal and gluteal (buttock) origins have also been documented. The time interval between primary tumor resection and pericardial metastasis ranges from 1 year to as long as 19 years (3–5).

A key diagnostic challenge lies in the non-specific imaging features of MLPS, especially when it involves atypical locations such as the pericardium. These features often overlap with those of benign lesions, leading to clinical misdiagnosis and treatment delays (3, 6, 7). Common mimickers and their imaging characteristics include hemangiomas, which may show very high T2 -weighted signal and progressive fill-in on delayed postcontrast imaging; cysts, which demonstrate very high T2-weighted signal without internal enhancement; and subacute to chronic hematomas, which can display variable T1/T2 signal with peripheral or septal enhancement. The imaging features of these benign lesions may overlap with those of myxoid or fat-containing sarcomas—particularly when the latter display smooth margins and a preserved fat plane. In this case, multimodal imaging provided a benign-leaning impression rather than a “non-diagnostic” one. However, because delayed fill-in and well-circumscribed morphology are not specific, malignant entities such as myxoid liposarcoma must remain within the differential diagnosis until confirmed by histopathology.

## Clinical data

**Figure 1:**
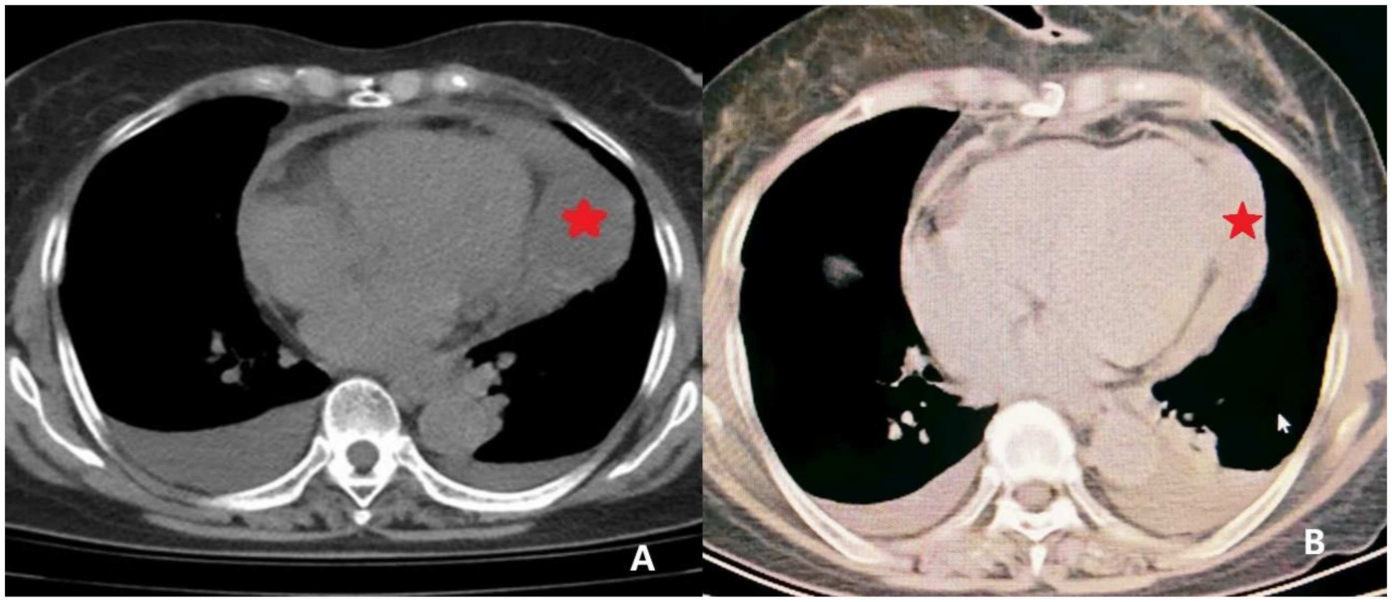
CT imaging of the pericardial mass. **(A)** Preoperative CT image revealing localized pericardial thickening with associated areas of fluid density. A focal soft tissue density is noted at the cardiac apex (indicated by *) concerning for a space-occupying lesion. **(B)** Postoperative CT image demonstrating resolution of the pericardial thickening and absence of the previously noted soft tissue density at the cardiac apex, indicating successful resection.

**Figure 2:**
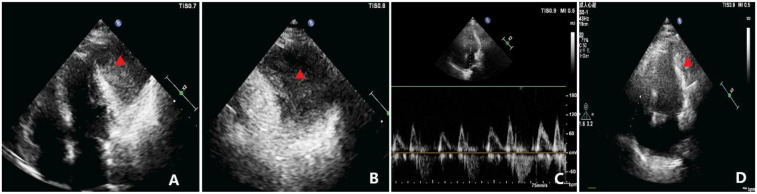
Echocardiographic findings. **(A)** Apical four-chamber view showing a homogenous mass, measuring approximately 69 × 50 mm, adjacent to the anterolateral wall of the left ventricle (indicated by the triangle), seemingly within the pericardial cavity. The mass is well-circumscribed, and the left ventricular anterolateral wall has mild hypokinesis. **(B)** Magnified view of **(A)** showing the relationship between the mass and adjacent structures in greater detail (indicated by the triangle). **(C)** Diastolic flow velocities across the mitral and tricuspid valves demonstrating significant respiratory variation, with the change in peak diastolic velocity during inspiration exceeding 30%. **(D)** Postoperative TTE apical view showing marked reduction in the residual mass burden compared with baseline (indicated by the triangle), with improvement in regional wall motion.

A 69-year-old woman presented with a 15-day history of chest tightness of unclear origin, which improved with rest but was accompanied by reduced activity tolerance. Chest CT (Figure 1) indicated localized thickening of the pericardium with a fluid-density shadow and a soft tissue-density lesion at the cardiac apex. Cardiac ultrasound (Figure 2) revealed left atrial enlargement and an echogenic mass (∼69 mm × 50 mm) in the pericardial cavity outside the anterior left ventricular wall. The lesion had relatively clear margins and was associated with slightly impaired ventricular wall motion, suggesting an extracardiac mass.

Medical history of the patient included intestinal polyps for 20 years, lower limb varicose veins, and a prior diagnosis of gluteal myxoid “liposarcoma”, 6 years earlier. Given this history, pericardial metastasis from the previous myxoid liposarcoma was considered the leading differential diagnosis.

**Figure 3:**
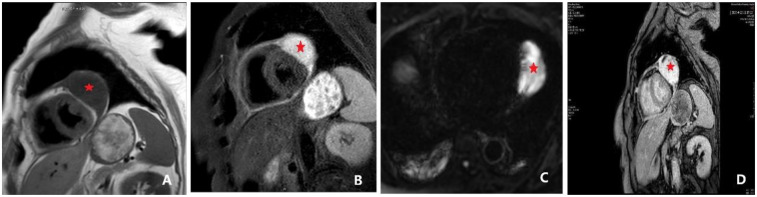
Cardiac magnetic resonance (CMR) findings, including late gadolinium enhancement (LGE). **(A)** Cardiac MRI revealing a space-occupying lesion within the pericardium, lateral to the left ventricle, suggestive of a hemangioma. The findings also included a thickened pericardium with a small amount of effusion. An ovoid lesion with abnormal signal intensity (indicated by *), measuring approximately 37 × 62 × 61 mm, was identified in this location. The lesion appeared hypointense on T1-weighted imaging (T1WI), and a discernible fat plane was present between the lesion and the lateral wall of the left ventricle. **(B)** Lesion appeared hyperintense on T2-weighted imaging (T2WI). **(C)** Lesion appeared hyperintense on both diffusion-weighted imaging (DWI) and apparent diffusion coefficient (ADC) maps. Following contrast administration, the lesion demonstrated marked heterogeneous enhancement, with progressive internal fill-in observed on delayed scans. **(D)** Late gadolinium enhancement (LGE) demonstrating progressive internal fill-in.

MRI (Figure 3) revealed enlargement of both atria and an oval abnormal lesion (∼37 mm × 62 mm × 61 mm) located laterally within the pericardium; the lesion showed high signal intensity on T2WI, low signal on T1WI, and high signal on DWI and ADC sequences. The lesion exhibited marked heterogeneous enhancement with delayed inward contrast filling, suggesting a possible hemangioma. Early (30–90 s) and late (3–10 min) postcontrast phases demonstrated progressive internal fill-in with delayed enhancement (LGE). Notably, T2-weighted fat-suppression imaging revealed no fat tissue-related signal loss.

Based on these imaging characteristics and clinical factors, including lesion size, persistent symptoms (chest tightness), proximity to the myocardium (with risk of functional impairment), and the prior sarcoma history of the patient, we recommended surgical resection over surveillance despite the benign-leaning imaging impression. It should be noted that the combination of clear margins and delayed internal enhancement is non-specific and can occur in both benign and malignant masses, necessitating correlation with histopathological findings.

**Figure 4:**
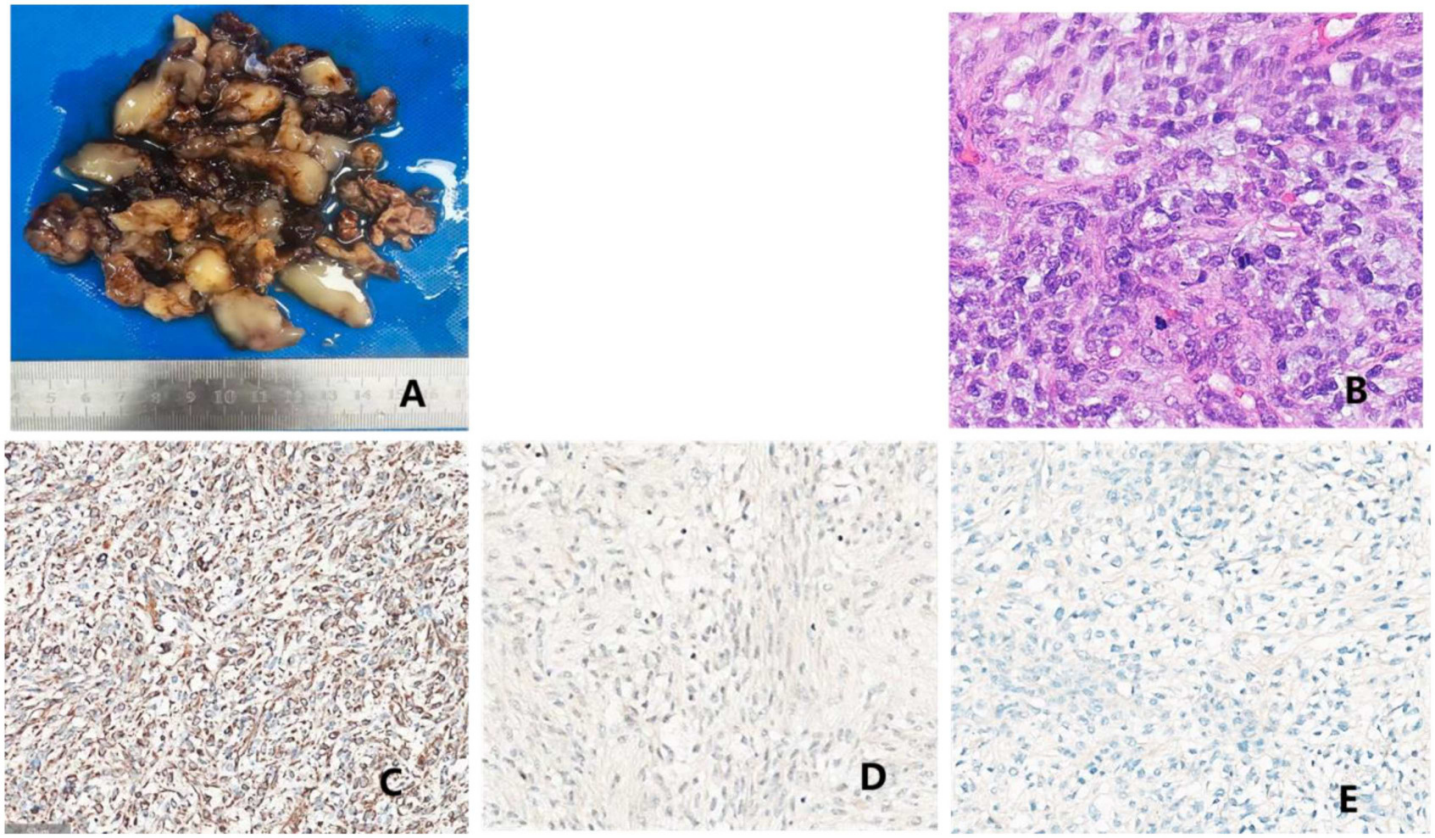
Pathological findings: **(A)** surgically resected pericardial mass comprising blood clots and gelatinous material, measuring a total volume of 16 × 8 × 1.5 cm. The cut surface predominantly appeared gelatinous, with localized gray-white areas showing slightly firm texture. Additionally, a small piece of gray-red cystic wall tissue (measuring 4 × 3 × 0.8 cm) was observed, whose inner surface was attached mainly with blood clot-like material, with a wall thickness of 0.2–0.3 cm. **(B)** Microscopic histopathological findings: spindle cell tumor with relatively uniform, short-spindle cells exhibiting cellular atypia and conspicuous mitotic figures, abundant myxoid stroma, and rich vascularity. Immunohistochemical staining results: **(C)** vimentin (+); **(D)** MDM2 (weak+); and **(E)** MUC4 (weak+).

Surgical excision revealed a gelatinous tumor (60 × 80 mm) at the diaphragmatic surface near the cardiac apex, with unclear boundaries to the visceral pericardium. Pathological examination (Figure 4) indicated a spindle cell tumor with myxoid stroma, prominent nuclear atypia, and frequent mitotic figures. Immunohistochemistry results revealed CK(−), vimentin(+), CD34(−), SMA(−), desmin(−), β-catenin (cytoplasmic+), CDK4(−), MDM2(weak+), MUC4(weak+), STAT6(−), S-100(−), and a Ki-67 proliferation index of ∼20%. The initial pathological diagnosis inclined toward myxoid fibrosarcoma; however, molecular pathology testing (Table 2) confirmed the presence of FUS-DDIT3 fusion gene, confirming the final diagnosis of myxoid liposarcoma. During postoperative follow-up, both thoracic CT and cardiac ultrasound demonstrated a significant reduction of the pericardial space-occupying lesion compared to preoperative findings, along with improvement in left ventricular diastolic function. PET-CT examination was not performed due to patient refusal. No evidence of local recurrence or distant metastatic lesions was observed during 9 months of systematic surveillance involving regular clinical assessments and serial imaging studies.

## Discussion

Myxoid liposarcoma is a subtype of soft tissue sarcoma, accounting for approximately 5% of such tumor cases (1). It typically occurs in the extremities (2), whereas involvement of the pericardial cavity is exceedingly rare and usually metastatic, with very few reported cases of primary pericardial myxoid liposarcoma (6, 8). Considering the prior gluteal tumor and the current pericardial presentation of the patient, this lesion is best interpreted as metastatic myxoid liposarcoma. The patient in this report exhibited the following notable characteristics: First, she was an elderly woman with a prior history of gluteal liposarcoma. Second, the tumor presented at an unusual location within the pericardial cavity, exhibiting imaging features markedly similar to hemangioma. Key imaging features in this case—including well-circumscribed margins and progressive delayed enhancement—were benign-appearing but non-specific. Importantly, a lack of overt myocardial infiltration should not exclude malignancy, as myxoid sarcomas can also present with smooth interfaces and delayed fill-in. Third, the tumor exhibited predominant spindle cell morphology, which differs significantly from the typical round or oval cells characteristic of liposarcoma. The immunohistochemical profile deviated from that of classical myxoid liposarcoma, instead resembling fibrosarcoma, thereby necessitating molecular analysis (FUS-DDIT3 fusion gene) for establishing a definitive diagnosis.

Analysis revealed several critical reasons underlying the misdiagnosis. First, inadequate integration of the clinical history played a pivotal role, particularly neglecting the diagnostic significance of previous gluteal liposarcoma of the patient. Recurrent liposarcoma can present notable histological variation, with a predominance of myxoid stroma, which in this case resulted in the initial misdiagnosis as myxoid fibrosarcoma. The fundamental principle of unified diagnosis, recognizing the possibility of recurrence or metastasis from a previous tumor, was overlooked. According to research by Muratori et al. (9), approximately 14% of patients develop metastases, with a notably high rate of extra-pulmonary metastasis (approximately 55%). Frequent metastatic sites include the retroperitoneum, abdominal wall, chest wall, and other fat-rich tissues or organs. Therefore, clinicians should think beyond the conventional view that these tumors primarily occur in the extremities and should include recurrence or metastasis in the differential diagnosis when evaluating tumors at rare locations, especially in patients with a history of soft-tissue sarcomas. Second, significant overlap in imaging features contributed to the misdiagnosis (Table 1). Accordingly, cardiac imaging reports should be framed as a structured differential, explicitly listing benign options (e.g., hemangioma, cyst, hematoma) and malignant possibilities (e.g., myxoid liposarcoma, angiosarcoma, metastasis), stating that delayed enhancement is shared across both categories. In this case, MRI findings closely resembled a hemangioma, with both lesions showing high T2WI signals and delayed enhancement. However, heterogeneous enhancement and delayed contrast filling could easily be misinterpreted as benign characteristics (10).

**Table 1:** Comparison of imaging characteristics.

Imaging modality	Sarcoma	Hemangioma	Findings in this case
MRI-TIWI	Often presents with a heterogeneous signal, typically iso- or hypointense, with indistinct margins and poor demarcation from surrounding tissue	Intermediate signal with well-defined margins	Hypointense
MRI-T2WI	Slightly hyperintense to hyperintense. The internal signal varies depending on its components	Hyperintense	Hyperintense
T2 fat-suppression sequence	Signal loss may occur if fatty components are present; no signal loss in lipid-poor subtypes	No signal loss	No signal loss (tumor was lipid-poor, confirmed by pathology)
DWI/ADC	Hypervascular lesions show significant enhancement; a “tail sign” (fascial infiltration) may be visible	Heterogeneous enhancement	Marked heterogeneous enhancement
Enhancement pattern	Heterogeneous enhancement (weak enhancement in myxoid areas, rapid wash-in and wash-out in solid areas)	Progressive fill-in enhancement (prominent in the delayed phase)	Progressive internal fill-in on delayed scans
Margins	Infiltrative (high-grade) or well-circumscribed (low-grade)	Well-defined, lobulated	Relatively well-defined
Fat plane	Liposarcomas may contain fatty components	Rare	A fat plane is visible between the lesion and the lateral wall of the left ventricle

Beyond individual cases, contemporary cohort data support the central role of CMR in evaluating suspected cardiac masses. In a multicenter outcome study (*n* ≈ 900), CMR showed ∼98% agreement with the final diagnosis and provided independent prognostic stratification; additionally, a long-inversion time LGE sequence reliably distinguished thrombus from tumor (11).

Complementary data from high-volume centers further show that CMR outperforms echocardiography in tumor typing and malignancy assessment, with malignant masses more often presenting as larger, infiltrative, and demonstrating LGE (12).

Recent editorial guidance synthesizes these findings into a pragmatic multimodality pathway: TTE for screening, comprehensive CMR for tissue characterization (including long-TI LGE), and selective CT/PET when CMR findings are equivocal. This approach aligns with our case and explains how benign-appearing features may still mask sarcoma (13). The absence of characteristic “curvilinear vessels” and “tail signs” (indicative of fascial infiltration and typically seen in myxoid liposarcoma) (3, 7, 14, 15) and preservation of the fat plane between the lesion and myocardium further led to an incorrect assumption of clear benign lesion boundaries. Moreover, the increased heterogeneity of the tumor complicated the pathological diagnosis (Table 2). Morphologically, the predominance of spindle cells and absence of typical adipocytes led to confusion with myxoid fibrosarcoma. Immunohistochemistry results were also conflicting; although CD34(−) and CDK4(−) matched typical myxoid liposarcoma characteristics, the negative S-100 staining differed from the usual S-100 positivity seen in myxoid liposarcoma, further complicating accurate identification. Reassuringly, the initial benign-leaning imaging diagnosis did not cause direct harm, as the team ultimately decided on surgical resection based on the tumor size, symptomatic impact, and the prior sarcoma history of the patient. No delays in treatment or adverse events were associated with the preliminary misdiagnosis.

**Table 2:** Histologic, immunohistochemical, and molecular findings.

Category	Feature/Marker	Myxoid liposarcoma	Myxoid fibrosarcoma	Findings in this case
Histomorphology	Cell morphology	Uniform round/oval lipoblasts, small, eccentric nuclei	Spindle cells, significant atypia, hyperchromatic, and pleomorphic nuclei	Spindle cells with atypia
	Stroma	Myxoid stroma + delicate “chicken-wire” capillary network	Myxoid + collagenized areas, abundant but irregular blood vessels	Myxoid stroma
	Mitosis	Usually few (low- to intermediate-grade)	Readily visible (intermediate- to high-grade)	Readily visible
	Necrosis	Rare	Can be present (high-grade)	
Immunohistochemistry	s-100	(+) Adipocytic differentiation	(−)	(−)
	MUC4	(−)	Partially (+)	Weak (+)
	CD-34	30%–50% focally (+)	50%–70% (+)	(−)
	MDM2	(−)	(−)	Weak (+)
	CDK4	Usually (−)	(−)	(−)
Molecular pathology	FUS-DDIT3	>90% (+)	(−)	(+)
	MDM2 amplification	(−)	(−)	
	FUS-CREB3L2/L1	(−)	Low-grade malignant type (+)	

This analysis of multimodal imaging misdiagnosis illustrates the significant diagnostic challenges associated with pericardial myxoid liposarcoma. Its substantial overlap with benign lesions on imaging, combined with marked histological heterogeneity, notably increases the risk of clinical misdiagnosis. To improve diagnostic accuracy, clinicians should particularly emphasize: first, comprehensive integration of clinical history, including thorough inquiry about the original pathological subtype in patients with a previous history of soft tissue sarcoma, and molecular pathology characteristics; second, optimization of functional imaging assessments, particularly focusing on lesion enhancement patterns and vascular features; and third, establishment of a multidimensional pathology diagnostic pathway that integrates morphological assessment, immunohistochemistry, and molecular analysis. This case emphasizes the necessity for multidisciplinary collaboration among cardiology, cardiothoracic surgery, radiology, pathology, and molecular diagnostics departments. In line with contemporary recommendations, a practical diagnostic pathway is TTE → comprehensive CMR (with long-TI LGE) → selective cardiac CT and/or FDG-PET when CMR remains equivocal or small/mobile masses are suspected (13). Practically, the key lesson from this case is that when malignancy cannot be reasonably excluded, an operability-adjusted early resection approach is preferable to prolonged observation. While improved multidisciplinary collaboration and refined imaging-pathology criteria can reduce misclassification, complete prevention remains unrealistic for rare cardiac tumors. When the clinical context or imaging findings raise concern for malignancy—or diagnostic uncertainty persists despite non-invasive work-up—expedited surgical excision should be considered over watchful waiting after a multidisciplinary risk–benefit assessment. Systematic follow-up—including PET-CT when appropriate—adds critical prognostic and staging information, reduces diagnostic uncertainty in rare cardiac sarcomas, and should be explicitly reported in case studies. Accumulating additional cases is required to refine diagnostic criteria for rare myxoid sarcomas in uncommon locations, thereby facilitating innovation and individualization of therapeutic strategies in clinical practice.

## Data Availability

The original contributions presented in the study are included in the article/Supplementary Material; further inquiries can be directed to the corresponding authors.
