# Supplementary figures and images for: Case report and diagnostic implications of misdiagnosis of pericardial myxoid liposarcoma by multimodal imaging
Source: Front Cardiovasc Med. 2025 Oct 9;12:1685844. doi: 10.3389/fcvm.2025.1685844 (PMC12546078; doi:10.3389/fcvm.2025.1685844)

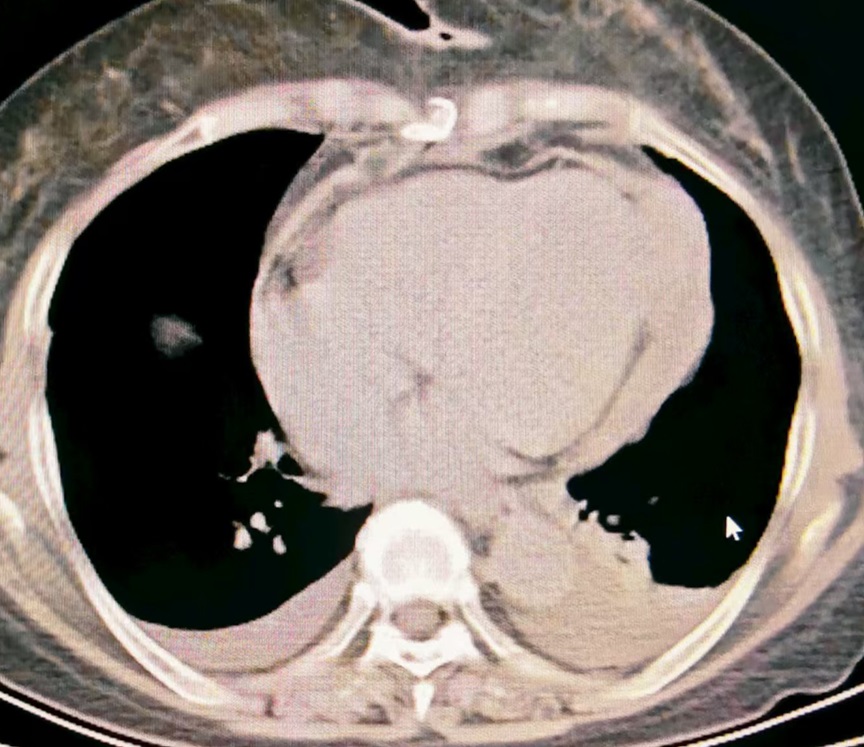

Supplement: Supplementary file 1 [file Image1.jpeg]

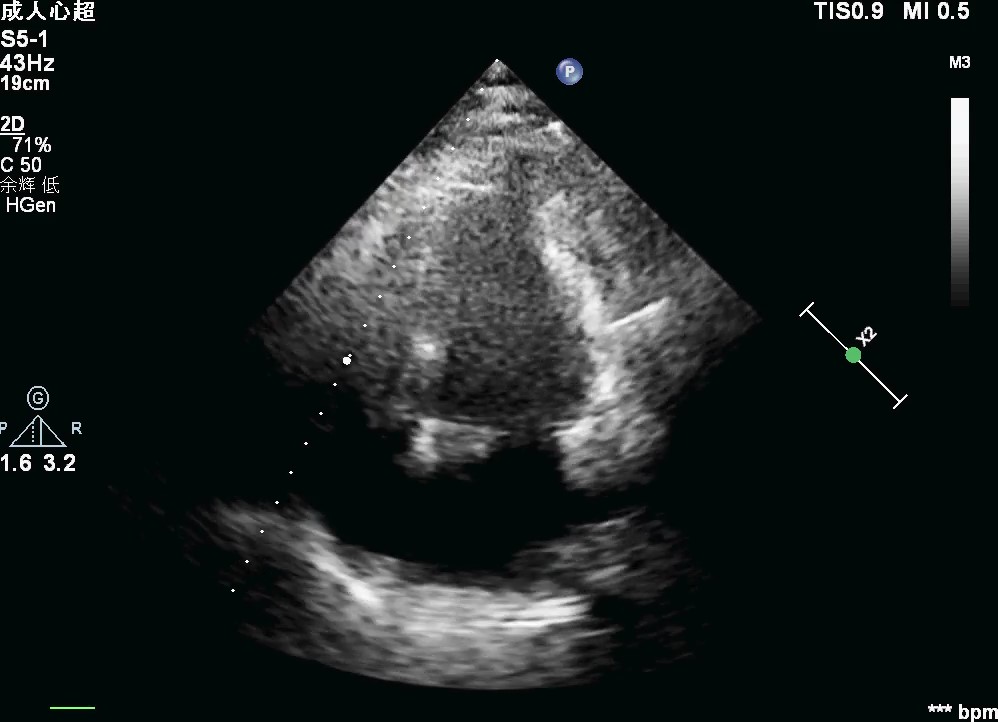

Supplement: Supplementary file 2 [file Image2.jpeg]

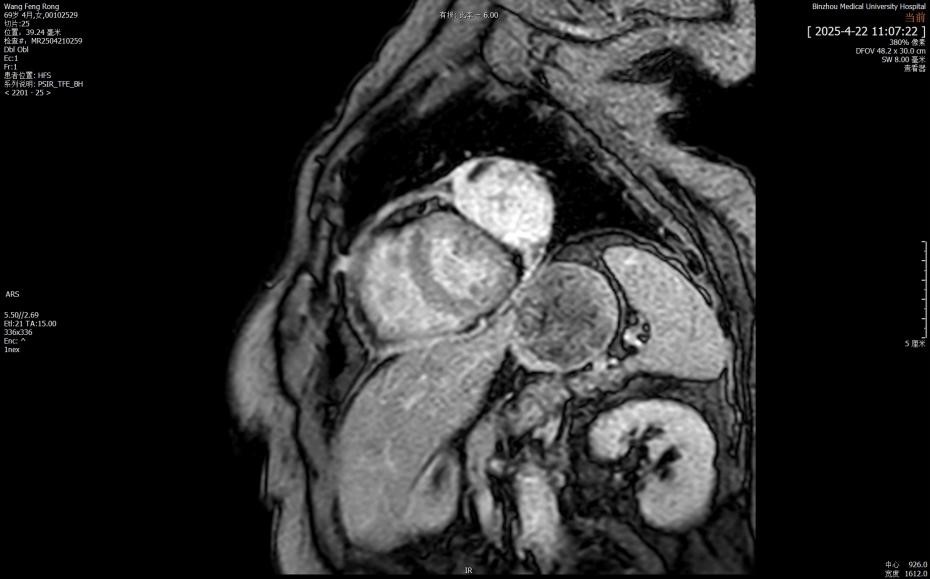

Supplement: Supplementary file 3 [file Image3.jpeg]
